# Supplementary material for: Exploring an Intervention to Enhance Positive Mental Health in People with First-Episode Psychosis: A Qualitative Study from the Perspective of Mental Health Professionals
Source: Healthcare (Basel). 2025 Jul 28;13(15):1834. doi: 10.3390/healthcare13151834 (PMC12346678; doi:10.3390/healthcare13151834)
Supplement: Supplementary file 1 [file healthcare-13-01834-s001.zip › Supplementary Material S1.pdf]

<u>Field Diary of Expert Group Sessions</u>

SUMMARY OF EXPERT GROUP SESSIONS – Tailored to the Mentis Plus FEP Program - Brief Version

Session 1

Date: March 2024

Duration: 1 hour 30 minutes

Participants present: Psychiatrist, two clinical psychologists, social worker, two mental health nurses.

1. Descriptive Observations

Initial meeting with the FEP multidisciplinary team at PSSJD. High engagement, collaborative tone. The group was open to innovation and emphasized tailoring the program to the emotional and motivational needs of people with FEP.

2. Methodological Observations

The session followed a free-flow discussion format. The facilitator introduced the goal of tailoring Mentis Plus, and participants brainstormed the best session structure for feasibility and engagement. Main methodological insight: reduce the number of sessions and simplify content without losing essence.

3. Key Contributions from the Group

Suggestion to reduce from 18 to 8 sessions. All sessions to be group-based. Maintain core SMP factors. Avoid content potentially triggering delusional ideation. Homework and structure must be simplified. Initial assessment tools agreed upon.

4. Facilitator’s Reflections

Participants provided grounded, clinically useful recommendations. There was consensus on prioritizing accessibility, emotional safety, and dynamic participation. Very positive start.

Session 2
Date: One week later

Duration: 1 hour 45 minutes

Participants present: Same expert team as Session 1

1. Descriptive Observations
Presentation of the first draft of the tailored program. The group followed closely, reading through each session's structure and content. Participants asked questions and suggested alternatives based on clinical experience.

2. Methodological Observations
Discussion organized by session. Each participant contributed suggestions for feasibility, clarity, and therapeutic safety. Feedback was specific and grounded in direct experience with the FEP population.

3. Key Contributions from the Group
Proposal to remove the mirror-in-hat exercise due to risk of triggering psychotic symptoms. Recommend replacing deep breathing with walking meditation or Jacobson's relaxation. Expand emotional reflection activities.

4. Facilitator's Reflections
Excellent level of detail and sensitivity from the group. Their feedback ensured psychological safety and therapeutic coherence. Proposed changes were coherent with the tailoring objectives.

Session 3
Date: Following week

Duration: 1 hour 30 minutes

Participants present: Experts from previous sessions.

1. Descriptive Observations
Final validation session. Revised version presented. The group agreed that the changes improved relevance and practicality. Final suggestions were made for closing activities and slogans.

2. Methodological Observations

Validation was structured: review of final sessions, assessments, and sequence. Final decisions made collectively. The group endorsed the revision and declared it ready for implementation.

3. Key Contributions from the Group

Added group closing ritual (personal slogan). Confirmed all sessions would be group-based. Approved final instruments (QSMP, satisfaction scale).

4. Facilitator's Reflections

The group showed strong alignment on the final structure. Validation felt solid and endorsed. All revisions align with initial program values and clinical best practices for FEP.
