# Supplementary material for: Exploring an Intervention to Enhance Positive Mental Health in People with First-Episode Psychosis: A Qualitative Study from the Perspective of Mental Health Professionals
Source: Healthcare (Basel). 2025 Jul 28;13(15):1834. doi: 10.3390/healthcare13151834 (PMC12346678; doi:10.3390/healthcare13151834)
Supplement: Supplementary file 1 [file healthcare-13-01834-s001.zip › Supplementary Material S2.pdf]

Overview of Revisions from the Original Mentis Plus to the Mentis Plus FEP Program - Brief Version

Original Mentis Plus Session	Mentis Plus FEP Brief Group Version	Maintained from Original	Removed from Original	Modifications and Additions
Initial	Initial	Presentation of participants and researchers.	Presentation of the SMP Program.<br>Assess SMP of participants with QSMP.	Assess literacy in SMP.<br>Sign informed consent.<br>Sociodemographic questionnaire.
1st Session (Individual)	-	Welcome.<br>Inform participants about the factors to be worked on.<br>Informed consent.<br>Sociodemographic questionnaire to classify participants.<br>Program next session.	-	-
F1-A	F1	Greetings and group introduction.<br>Write interventions from emergencies with positive memories.	Greetings and group introduction.<br>End session	Write interventions from emergencies with positive memories.<br>Share with the group.

		Share with the group.<br>Discussion on which emotions are associated with positive memories.<br>Homework: Write down positive things that happened during the week.<br>End session and plan next one.	and plan next one.	Discussion on which emotions are associated with positive memories.
F1-B	F1	Initial greetings.<br>Verify homework (list of positive things during the week).<br>Write down a flaw.<br>Share with the group.<br>Write a virtue next to the flaw.<br>Share with the group.<br>Discussion about feelings when replacing flaws with virtues.<br>Program next session and final greetings.	Initial greetings.<br>Write a flaw.<br>Share with the group.<br>Write a virtue next to the flaw.<br>Share with the group.<br>Discussion about feelings when replacing flaws with virtues.	Verify homework (list of positive things during the week).

F1-C	-	Initial greetings. Hat mirror exercise. Discussion space. Program next session and final greetings.	Not performed.	-
F2-A	F2	Initial greetings. Play 'Sentir' by Luz Casal. Reflect on the message it conveys. Write down things we want to change in our lives for others' benefit. Share with the group. Discussion on emotions. Homework: Write down concrete strategies to modify life parameters. Program next session and final greetings.	Initial greetings. Play 'Sentir' by Luz Casal. Reflect on the message it conveys. Write down things we want to change in our lives for others' benefit. Share with the group.	
F2-B	-	Initial greetings. Share homework (concrete	Not performed.	-

		strategies for life change). Balloon exercise (things to help others). Reflection on activity. Reflect on the importance of helping others. Homework: Specify an action to help others. Program next session and final greetings.		
F2-C	-	Initial greetings. Share homework about actions to help others. Reflect on them. Discussion space. Program next session and final greetings.	Not performed.	-
F3-A	F3	Initial greetings. Write 2-3 recent situations with strong emotions or impulsiveness, including a situation where feelings were	Initial greetings. Write 2-3 recent situations with strong emotions or impulsiveness,	

		expressed well and one where they were not. Share with the group and express differences between the two. Deep breathing technique. Avoidance technique training. Discussion space. Program next session and final greetings.	including a situation where feelings were expressed well and one where they were not.	
F3-B	-	Initial greetings. Cognitive techniques for emotional contro.l Adapt these to individual situations. Discussion space. Program next session and final greetings.	Not performed.	-
F3-C	-	Initial greetings. Explain a recent emergency intervention. Encourage positive feedback. Teach relaxation	Not performed.	-

		strategies. Teach autogenic relaxation technique by Schultz. Discussion space. Encourage practicing at home. Program next session and final greetings.		
F4-A	F4	Initial greetings. Promote verbalizing feelings, perceptions, and fears. Positive feedback. Teach participants to value principles instead of following feelings (self-regulation). Discuss the consequences of not facing responsibilities. Encourage independence of judgment and personal safety. Complete the		

		'self-autonomy' table. Reflect on what is important to achieve and what they really want to achieve. Present the table to the group. Discussion space. Program next session and final greetings.		
F4-B	F4	Initial greetings. Ask what self-confidence is. Discussion space. SWOT technique (each participant thinks about their strengths, opportunities, weaknesses, and threats). Each participant shares analysis with the group. Discussion space. Program next session and final greetings.	Initial greetings. Ask what self-confidence is. Discussion space. SWOT technique (each participant thinks about their strengths, opportunities, weaknesses, and threats). Each participant shares analysis with the group.	

F4-C	F4	Initial greetings. Each participant thinks of 5 things they have achieved in life and writes them down. Each participant reads their list. Suggest that they keep the list to consult when in doubt about their abilities. Discussion space to express feelings and share. Program next session and final greetings.	Initial greetings. Each participant thinks of 5 things they have achieved in life and writes them down. Each participant reads their list. Suggest that they keep the list to consult when in doubt about their abilities.	
F5-A	-	Initial greetings. Maier's threads exercise. Discussion space. Homework: Mobilize physical and mental resources to solve problems and supervise progress. Program next	Not performed.	-

		session and final greetings.		
F5-B	F5	Initial greetings. Share homework of mobilizing physical and mental resources for problem-solving. Reflect on progress achieved. Evaluate in group if the strategy is adequate. Present problem-solving wheel. Encourage using it. Discussion space. Conclude that every problem is unique and the solution is in each person. Program next session and final greetings.	Initial greetings. Present problem-solving wheel. Encourage using it. Discussion space. Conclude that every problem is unique and the solution is in each person.	
F5-C	-	Initial greetings. Each participant completes the phrase “I think” or “I want”.	Not performed.	-

		Then they complete the phrase “I achieve”. Discussion space. Conclude that each response is individual. Program next session and final greetings.		
F6-A	F6	Initial greetings. 'Relations box' exercise: Write down anonymously a difficulty faced in their professional practice, fold it, and put all papers in a box. Each participant randomly picks a paper from the box and reads it anonymously. Each person suggests a solution individually. Do the same for the other papers and participants until everyone	Initial greetings. 'Relations box' exercise: Write down anonymously a difficulty faced in their professional practice, fold it, and put all papers in a box.	

		reads one. Discussion space. Program next session and final greetings.		
F6-B	-	Initial greetings. Dynamic printed questions. Discussion space. Homework: Make a list of 10 things that could happen if you do not work on your interpersonal relationships. Program next session and final greetings.	Not performed.	-
F6-C	-	Initial greetings. Share homework on 10 things that could happen if you do notwork on interpersonal relationships. Dynamic on 'cornerstones of professional relationships'. Discussion space.	Not performed.	-

Final	Final	Group reflection on the program. Sociodemographic final questionnaire. Administer QSMP. Thank participants. Collect feedback on the SMP program. Provide guidance for follow-up. Close program.	Final session conducted in a group, not individually. Administered QSMP and satisfaction questionnaire. Group reflection with a final dynamic activity: create a personal slogan to summarize what each participant takes away from the group. End group with an invitation for future contact if needed.	Program completion with group slogan and reflection.
