# Supplementary material for: Exploring an Intervention to Enhance Positive Mental Health in People with First-Episode Psychosis: A Qualitative Study from the Perspective of Mental Health Professionals
Source: Healthcare (Basel). 2025 Jul 28;13(15):1834. doi: 10.3390/healthcare13151834 (PMC12346678; doi:10.3390/healthcare13151834)
Supplement: Supplementary file 1 [file healthcare-13-01834-s001.zip › Supplementary Material S3.pdf]

Mentis Plus FEP Program - Brief Version

Factors of Positive Mental Health (PMH):

- Personal satisfaction
- Prosocial attitude
- Self-control
- Autonomy
- Problem-solving and self-actualization
- Interpersonal relationship skills

Sessions of the Mentis Plus FEP Brief Group Version:

Initial session:

- Presentation of the participants, the program, and PMH (explaining each of the factors).
- Self-administered assessment of the PMH level (PMH Dr. Lluch questionnaire) present at the time of the assessment.
- Agree on the group day and attendance.
- Sign the CI and fill out the sociodemographic variables sheet for each participant.

First Session: Working on the Personal Satisfaction Factor:

- Explain what personal satisfaction is.
- Write on a paper a defect that characterizes you and share it.
- Then, next to the defect, write a virtue that characterizes you and share it.
- Discussion space about what participants felt when replacing defects with virtues.
- List of positive things that happened to them during this week and share it.

- Activity: share three gratitudes and three achievements. Propose to do it every night in writing.

Second Session: Working on the Prosocial Attitude Factor:

- Evaluate the activity of noting three gratitudes and three achievements performed at home daily.
- Explain what a prosocial attitude is.
- Play the song "Sentir" by Luz Casal and reflect on the message it conveys and what the song makes them feel.
- Then, ask each person to express what they would like to change in their life that includes the well-being of others (thus developing a helping attitude for the community).
- Write concrete strategies to achieve this and propose taking the first step of the first objective for the next week.

Third Session: Working on the Self-Control Factor:

- Evaluate the activity of taking the first step of the first objective proposed in the previous session.
- Explain what self-control is.
- Ask each person to write down two recent situations in which they felt a strong emotion or impulsivity, including a situation in which they consider they adequately conveyed their feelings and one in which they consider they did not convey them adequately.
- Then, each person shares the described situations and expresses the difference they see between them.
- Explain the STOP technique (silence, time, observe, and action plan) to work on impulsivity.
- Teach the relaxation technique in movement, share opinions, and propose regular practice until the next session.

Fourth Session: Working on the Autonomy Factor:

- Evaluate the regular practice of the relaxation exercises proposed in the previous session.
- Explain what autonomy is.
- Write down the strengths, opportunities, weaknesses, and threats of each person using the SWOT technique and share it.
- Explain the wheel of emotions, provide one, as well as an emotional diary guide (situation, thought, emotion, what I do, and how it ends/solves).
- Then, each person must write down five things they have achieved during their life and share them with the group.
- It is recommended to keep this list for those moments when they doubt their own abilities.

Fifth Session: Working on the Problem-Solving and Self-Actualization Factor:

- Explain what problem-solving and self-actualization are.
- Present a problem they have recently encountered and explain how they solved it.
- Other people comment on how they would have done it or if something similar has happened to them and how they solved it, and they present different ways to solve it.
- Then, explain the problem-solving wheel and apply it to the situations mentioned earlier.
- Apply it to other situations that arise and encourage its use to evaluate the experience in the next session.

Sixth Session: Working on the Interpersonal Relationship Skills Factor:

- Evaluate the experience of having applied the problem-solving wheel to situations experienced last week.
- Explain what interpersonal relationship skills are.

- Write on a sheet a difficulty the person encounters in their daily life, fold it, and put them all together in a box.
- Each person will take a random sheet from the box and, anonymously, read what it says, and each person will have to give a possible solution individually. The same will be done with the other sheets and participants until everyone has read one.
- Then, open a discussion space about what they felt.

Final session:

- Group reflection on the program.
- Evaluate the PMH level present at the time of the assessment (PMH Lluch questionnaire) and the degree of personal satisfaction with the program (ad hoc scale).
- Dynamic of creating a small and striking slogan of what each person takes from the group in summary.
- Finally, the program is closed.

Each session will last **one hour**.

Groups will consist of **6-8 people**.

Intervention:

Based on the Mentis Plus Positive Mental Health Program for individuals with FEP, brief group version.
